# Rich autobiographical memory recall benefits from both novelty and similarity to other daily experiences

**DOI:** 10.21203/rs.3.rs-7402294/v1

**Published:** 2025-09-03

**Authors:** Erin Welch, Victoria Schelkun, Camille Gasser, Kathryn Lockwood, Lila Davachi

**Affiliations:** Columbia University; Columbia University; Columbia University; Temple University; Columbia University

## Abstract

Much existing laboratory research has shown that both novelty and prior knowledge benefit episodic memory, however they do so through differing mechanisms. Critically, autobiographical experiences are rarely completely novel or congruent with prior experience, existing somewhere within this spectrum of ‘absolute’ novelty to ‘absolute’ congruency. A prospective real-world autobiographical event sampling study was conducted to investigate this spectrum. We analyzed different types of novelty and how they predicted different episodic memory outcomes. We found that, although events that were participant-labeled as being ‘new’ were later remembered with greater vividness compared to ‘periodic’ and ‘routine’ events, ‘new’ events that were more semantically *similar* were recalled with the greatest vividness (what we are calling the ‘something old, something new’ principle). Further, participant-labeled novelty and relative semantic similarity had differing effects on the emotions associated with each event, the former increasing arousal-related emotions while the latter supported positive and minimized negative emotions. Finally, relative *emotional* distinctiveness predicted greater vividness and episodic detail at recall. These results suggest that different sources of novelty (i.e., participant-labeled novelty, semantic novelty, and emotional novelty) produce different effects on autobiographical memory and emotion, and that ‘maximal’ novelty may not lead to the greatest episodic recall later on.

## Introduction

What we remember can shape who we are, who we become, and how we measure the life already lived. For this reason, understanding which experiences become memorable holds considerable weight. Prior, majorly lab-based work has demonstrated that unique experiences tend to be more accessible in memory [1–9]. However, day-to-day life outside of the laboratory is complex. The novelty of a personal experience can stem from various sources and exist along a continuous scale of magnitude [10]. Further, congruency with prior knowledge, which is traditionally placed in opposition to novelty, has also been shown to benefit memory [11–16]. In order to better understand these interactions, it is important to tease apart how different types and degrees of novelty impact memory for autobiographical experiences.

Novel events are typically distinct, effectively ‘standing out’ in an overarching context. The ‘isolation effect’ demonstrates that such relative distinctiveness is more likely to be later recalled [1]. Novel events attract additional attention, increasing the likelihood of effective encoding and later memory for that information [4, 7]. Novelty can also elevate arousal, triggering the release of neuromodulators such as dopamine and norepinephrine, which facilitate long-term potentiation, a key mechanism underlying long-term memory formation [3, 5–9]. By contrast, information that conforms to our expectations can also endure in long-term memory. Animal work found that new paired associates were learned more rapidly when there was a pre-existing schema [12]. A wealth of behavioral data in humans has shown that encoding new information related to existing knowledge leads to rapid integration of such information into the current schema, thus making it more resistant to forgetting [11, 13–16]. To accommodate for these disparate observations, a ‘U-shaped’ function of congruency has been proposed and further experiments were conducted to show that information that was surprising and information that conformed to expectations both had higher recognition and recall rates compared to unrelated information [17, 18].

With this foundational work in mind, a subsequent question is how this U-shaped congruency effect translates to memory for complex, personal events. Recent work on real-world autobiographical memory has shown that a metric of daily ‘uniqueness’, composed of different measures collected during each day and during the recall test, was associated with greater recalled episodic details for older adults [19]. In the current study, we conducted a prospective real-world autobiographical ‘daily diary’ study, where at the end of our two-week sampling period (and after an additional two-week delay) young adults were given a memory recall test. Based on these daily participant reports, we found a significant effect of event novelty on memory vividness [20]. Events labeled as ‘new’ during the sampling period were later remembered with greater vividness after a two-week delay compared to events labeled as ‘periodic’ and ‘routine’. Further, ‘routine’ events were remembered with the least episodic detail at recall. Thus, it is clear that there is a positive effect of novelty on the richness of autobiographical memory recall. However, most real-world events are neither purely novel nor purely congruent. For example, grocery shopping in a new country (i.e., a familiar event in a new context) or encountering a celebrity during a routine commute (i.e., a novel event in a familiar context) each involve schema-congruent and novel components. These ‘intermediate’ experiences (i.e., events that include both novel and schema-congruent elements) do not fit neatly at either end of the U-shaped congruency curve. Recent work [10] proposes a gradient theory of schema and novelty, and suggests that the fate of a memory is influenced by the interplay between novel and congruent elements within that complex experience. An additional, critical consideration is that autobiographical experiences can comprise various *types* of novelty beyond event regularity-based judgements (e.g., novelty in *semantic* or *emotional* content). There is much to be investigated regarding how the interplay between novelty and congruency, as well as the specific type or types of novelty present, impact autobiographical memory richness. Little is known about whether different types of novelty have differing impacts on later memory.

To address these open questions, we leveraged our event dense-sampling daily diary study in young adults [20] to investigate the impact of novelty, congruency, and their interactions on later episodic recall of the reported autobiographical events. Importantly, we also investigated whether different sources of novelty (based on event regularity, semantic content, and emotional content) produced differing effects on our episodic memory outcomes.

## Methods

### Participants

A total of 51 participants (ages 18–35) were recruited and enrolled in this study from the Columbia University community. All participants lived in the United States and indicated that they were not currently diagnosed with any neurological or psychiatric disorder. Ten participants were withdrawn from this study because they missed two or more daily diary entries during the two-week daily survey. Thus, our final sample size is 41 participants (*M*_*age*_ = 25.6; *SD*_*age*_ = 4.2; 82.9% female). Participants were compensated $12/hour and informed consent was obtained from all participants. The research protocol was reviewed and approved by the Institutional Review Board of Columbia University and the study was conducted in accordance with relevant guidelines and regulations.

### Procedure

#### Overview.

**Figure 1:**
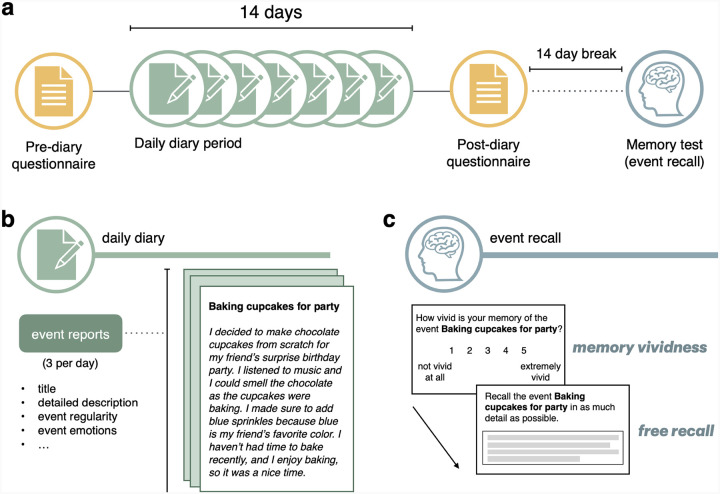
a) Experimental Design. The two-week daily diary period was flanked by a pre-diary study questionnaire and a post-diary study questionnaire. Two weeks after the post-diary questionnaire, participants completed a memory test for the events that were reported during the daily diary period. b) Overview of relevant variables collected with each daily diary report (see Methods for details). Importantly, participants reported three events each day and generated a unique title after each event description (the title and description pictured above do not represent a true example from the dataset to maintain participant privacy). c) Illustration of the relevant memory test: event recall. Note that all events, whether they were part of the event recall test or the temporal memory test (see Methods for details), had corresponding subjective memory vividness ratings.

Participants completed daily questionnaires via Qualtrics on their smartphones or computers spanning a 4-week period. The study consisted of four phases: the pre-survey questionnaire period, a two-week daily diary period, the post-study questionnaire period, and the long-term memory test (Fig. 1). The current study is focused on the daily diary collection and memory test phases. Thus, data from the pre- and post-study surveys are not reported here.

#### Daily diary period.

During the daily diary period, participants completed an identical survey each day for two weeks (14 days). They were instructed to complete the survey at any point between receipt of the survey link (sent ~ 5 pm each day) and before they went to bed. The survey began with general questions regarding the participant’s thoughts, feelings, and actions during that day. Next, they were asked to report and describe **three specific events** that took place that day. The task instructions identified an event as being ‘a thing you have done (e.g., listening to music, working on a project, talking on the phone, etc.) either on your own or with others’. Participants typed detailed descriptions of each event and provided a brief, distinct title for each event. For each event they were also asked about its objective and subjective duration, when during the day it took place, other people who were present (if applicable), how often the participant had experienced that type of event, how memorable and meaningful the event was, what emotions were evoked while experiencing the event, and what took place immediately before and after the event. In some cases, participants missed one (*N* = 13) or two (*N* = 3) days. In one instance a participant completed one extra daily diary day (15 instead of 14 days). Therefore, a range of 39–45 events were reported across participants for a total of 1,673 events.

#### Long-term memory test.

A memory test was administered approximately two weeks after the conclusion of the last daily diary entry (range: 14–19 days) and consisted of both event recall and temporal memory tests. One third of the events reported during the daily diary period (*M* = 13.8 events), one per day, was randomly selected to be included in the event recall test, the remaining two-thirds (*M* = 13.2 pairs) were included to create event pairs for the temporal memory test. For the event recall test, participants were shown their own generated event titles to serve as memory cues. They were first asked to rate subjective vividness with which they could recall the corresponding event and then to freely recall (via typing) the event in as much detail as possible. Memory for other event details was then tested including the location and time of day, others who were present (if applicable), how long the event lasted, and what they were doing immediately before and after the event. Each event (*N* = 1,673) had a memory vividness rating regardless of whether it was part of the event recall or temporal memory test, but only one-third of the total events had a description at recall (*N* = 565). Therefore, it should be noted that analyses that include information about the event recall descriptions are based on that subset of events.

### Measuring event novelty during the daily diary period

**Figure 2:**
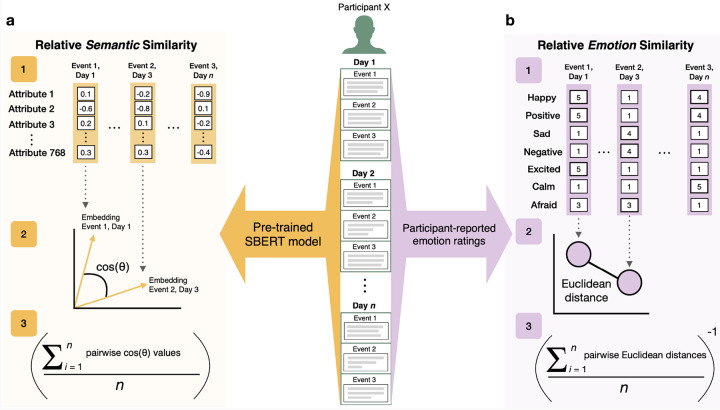
Calculating relative semantic similarity and relative emotion similarity. a) Illustration of how semantic embeddings were extracted and compared based on the reported daily diary events of one participant (‘Participant X’). b) Illustration of how emotion vectors were extracted and compared based on the reported event-level emotion ratings of one participant (‘Participant X’).

Event novelty was assessed using different measures. The first novelty-related variable of interest is *event regularity*. During the daily diary phase of the study, participants were asked to label each reported event as being ‘new’ (something they had never done before), ‘periodic’ (something they do occasionally), or ‘routine’ (something they do almost every day). This targets the participant’s own judgment regarding the novelty (or familiarity) of the reported event. Event regularity was treated as a categorical variable in all analyses to avoid assuming linear relationships. Overall, participants reported 359 ‘new’ events, 1,050 ‘periodic’ events, and 264 ‘routine’ events. Second, we used a natural language processing (NLP) model to quantify the *relative semantic similarity* (RSS; *M* = 0.31, *SD* = 0.09) between all reported events within each participant. Specifically, we used a pre-trained Sentence Transformer (SBERT) model (‘all-mpnet-base-v2’) that analyzes paragraph-level documents [21], in this case, diary entries reported during the two-week daily diary phase. This model outputs an embedding that can then be compared to the embeddings of other reported events. This embedding represents a vector in multidimensional semantic space, the multidimensionally representing a series of attributes that were set by the pre-trained model (in the case of the ‘all-mpnet-base-v2’ model, there are 768 dimensions). Cosine similarity values, each value representing the angle distance between two vectors in this multidimensional semantic space, were extracted (see Fig. 2a). The pairwise cosine similarity values were then averaged across all events within each participant, creating an event-level variable that represents the RSS of an event relative to all other events reported by that participant.

We also developed a metric of event emotion distinctiveness as a potential source of novelty (Fig. 2b). After providing event descriptions during the diary period, participants were asked to rate the intensity of seven feelings (‘happy’, ‘positive’, ‘sad’, ‘negative’, ‘excited’, ‘calm’, and ‘afraid’) related to the experience of each event on a scale of 1 to 5 (1 being ‘not at all’, and 5 being ‘a great deal’). This series of ratings were treated as event-level emotion vectors. In order to capture the magnitude of the differences between emotion vectors, Euclidean distances between an event’s emotion vector and every other event-level emotion vector from one participant was calculated. These pairwise distances were then averaged within participant, each averaged value representing the relative distinctiveness of the emotion ratings for that event compared to all other events reported by that participant. The inverse of this variable was then calculated to create a *relative emotion similarity* (RES) variable (*M* = 0.34, *SD* = 0.11), representing the relative similarity of an event’s emotion ratings relative to the emotion ratings of the remaining events reported by the participant. This was done so that both RSS and RES represent similarity (and novelty) with the same directionality. That is, both an increase in RSS and an increase in RES suggest greater relative event similarity, and less event novelty. Both RSS and RES were quantified using custom Python scripts, importing packages from the ‘sentence-transformers’ [21] and ‘scikit-learn’ libraries [22].

### Measuring memory vividness, detail, and stability for individual events

During the memory test, participants were presented with their self-generated event titles from the survey phase and were first asked to rate the vividness of their memory (using a scale from 1 to 5; 1 being ‘not at all’ and 5 being ‘highly vivid’). We measured memory for each event using two additional methods. First, we used a modified version of the Autobiographical Interview scoring approach [23]. Episodic detail counts consist of event (describing what happened, who was present, etc.), place (describing the event location), time (describing when the event took place or how long it lasted), thought/emotion (describing the feelings or thoughts of the self or others), and perceptual (describing sensory experience) details. In order to do this, we developed a novel method using large language models (LLMs). Specifically, we iteratively developed a GPT-4 prompt that outlined instructions to parse each event description into meaningful chunks, and then to subsequently categorize each chunk into episodic or non-episodic categories.

To validate this method of scoring, three independent human raters also segmented and categorized a subset of the event descriptions (*N* = 657 events; 30% of the full sample), following similar instructions that were inputted into GPT-4, guided by the autobiographical scoring methods mentioned previously [23]. We found a strong correlation when comparing episodic detail counts (based on the same subset of events) from human raters and GPT-4 (*r* = 0.71, *p* < 0.001), validating the assumption that GPT-4 would produce episodic detail counts that were comparable to manual, human-based scoring. Although episodic detail counts were extracted for both event descriptions during the daily diary period and subsequent event recalls during the memory test, only episodic detail counts at recall are included in these reported analyses.

Finally, for the subset of events that had corresponding event recalls during the memory test phase (about one-third of events) the semantic similarity between the description reported at survey and the subsequent recall was analyzed using SBERT to create a *memory stability* variable, each value representing the cosine similarity of a description at survey compared to its corresponding recall.

### Statistical analysis

The data were analyzed using Frequentist multilevel regression modeling with person-specific random intercepts and slopes. In cases where a full random effects structure (both random slopes and random intercepts) produced singular fits or convergence issues, random slopes were removed from the model. To verify the significant effects found in these ‘random-intercept-only’ models, corresponding Bayesian regression models that included the full random-effects structure were run. Reported significant Frequentist effects from random-intercept-only models were validated by meaningful Bayesian effects (see Supplementary Materialsfor more details). Given that a majority of the reported multi-level models used the random-intercepts-only structure (in most cases, to avoid possible overfitting based on singularity warnings), all results reported are based on this structure unless specified otherwise.

Analyses were conducted using custom analyses scripts in RStudio (ver 4.3.1). For data visualization, functions from the *ggplot2* (ver 3.5.1) package [24] were used. Frequentist regression models were computed using the *lme4* (ver 1.1–35.1) and *lmerTest* (ver 3.1.3) packages [25, 26]. The *parameters* (ver 0.22.2) package [27] was used to calculate the confidence intervals from these models. The *emmeans* (ver 1.10.0) package [28] was used when models included a three-level categorical predictor variable (i.e., event regularity), extracting estimated marginal means from each level, and then subsequently running pairwise contrasts between these estimates. The p-values from these estimated marginal means models were adjusted using Tukey’s method for comparing a family of estimates [29]. Bayesian models were run using the *brms* (ver 2.20.4) package [30].

When analyzing the effects of event regularity, RSS, and RES on the memory measures, the predictors were included together. Specifically, these models included the interaction between RSS and event regularity, and the additional predictor of RES. Post-hoc ANOVAs were run to verify that these models better explained the variance in the dependent variables (i.e., vividness ratings, total number of episodic details at recall, and memory stability) compared to reduced models (see Supplementary Materials for more details).

## Results

### Participant-labeled novelty predicts greater episodic memory: vividness, recalled episodic detail, and stability

The main effects of event regularity have been described in a separate manuscript [20]. These analyses showed that ‘memory vividness’ ratings during recall were significantly higher for ‘new’ compared to ‘periodic’ and ‘routine’ events. Further, ‘routine’ events were recalled with significantly less episodic detail compared to ‘new’ and ‘periodic’ events. These findings demonstrate the significant impact of novelty on autobiographical memory recall and inspired the focus of this current manuscript, which is to look for interacting effects across one’s own experiences with respect to semantic similarity (RSS) and emotional similarity (RES), while also including objective analyses of memory stability across time.

**Figure 3:**
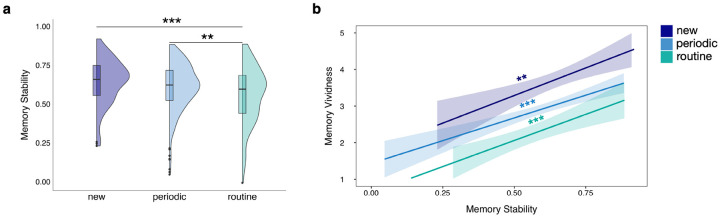
Effects of event regularity on memory stability and memory stability on memory vividness. a) The half-violin plots represent the overall distributions of memory stability values at each event regularity level. The boxplots within the half-violin plots span from the first to the third quartiles of the data, the horizontal lines representing the within-event regularity level median values. *p_tukey_<0.05, **p_tukey_<0.01, ***p_*tukey*_<0.001. b) Relationships between memory stability and memory vividness at each event regularity level. The lines and shaded bands represent linear regression lines and 95% confidence intervals. *p<0.05, **p<0.01, ***p<0.001.

We first examined the relationship between event regularity and memory stability, memory stability being the semantic similarity between a participant’s description during the diary phase and the corresponding long-term memory recall (Fig. 3a). We found that ‘new’ and ‘periodic’ events showed significantly greater memory stability compared to ‘routine’ events (*b* = 0.08, *SE* = 0.02, *CI* = [0.04, 0.12], *p*_*tukey*_ < 0.001; *b* = 0.06, *SE* = 0.02, *CI* = [0.02, 0.09] *p*_*tukey*_ = 0.006, respectively). There was no significant difference when comparing ‘new’ to ‘periodic’ events (*b* = 0.02, *SE* = 0.02, *CI* = [−0.01, 0.06] *p*_*tukey*_ = 0.306). We also found significant effects of memory stability on memory vividness (Fig. 3b), with greater memory stability across time predicting significantly greater memory vividness during recall, both when collapsing across event regularity level (*b* = 2.90, *SE* = 0.36, *CI* = [2.20, 3.60], *p* < 0.001), and when analyzing each event regularity level independently (‘new’: *b* = 2.48, *SE* = 0.80, *CI* = [0.90, 4.06], *p* = 0.002; ‘periodic’: *b* = 2.55, *SE* = 0.46, *CI* = [1.65, 3.45], *p* < 0.001; and ‘routine’: *b* = 2.72, *SE* = 0.75, *CI* = [1.25, 4.19], *p* < 0.001). Greater memory stability also predicted significantly more episodic details at recall (based on a multi-level model with random intercepts and random slopes) both when collapsing across event regularity level (*b* = 3.98, *SE* = 0.74, *CI* =[2.53, 5.44], *p* < 0.001), and within each event regularity level (‘new’: *b* = 4.26, *SE* = 1.51, *CI* = [1.29, 7.22], *p* = 0.005; ‘periodic’: *b* = 3.58, *SE* = 0.88, *CI* = [1.84, 5.32], *p* < .001; ‘routine’: *b* = 2.89, *SE* = 1.43, *CI* = [0.09, 5.69], *p* = 0.043). However, for the effect of memory stability on episodic detail for ‘routine’ events, it should be noted that only approximately 94% of the posterior slopes were positive when running a full-random-effects Bayesian model, thus falling short of the 95% cut-off (see Supplementary Materials for more details). The content of memory recall was a better match to the original event description during the daily diary phase when the event was ‘new’ or ‘periodic’, compared to a ‘routine’ event. Further, memory stability was associated with other aspects of the memory recall, greater memory stability predicting greater memory vividness ratings and more episodic details. The relationship between memory stability and memory vividness is particularly insightful, linking the participant-reported, subjective sense of memorability (i.e., vividness) to a more objective measure of memory reproducibility (i.e., memory stability).

### Relative semantic distinctiveness modulates the effect of event regularity on memory vividness

**Figure 4:**
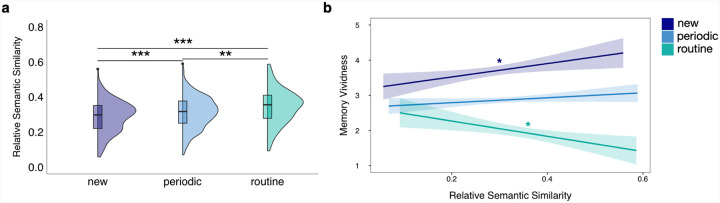
Effects of event regularity on relative semantic similarity and relative semantic similarity on memory vividness. a) The half-violin plots represent the overall distributions of relative semantic similarity values at each event regularity level. The boxplots within the half-violin plots span from the first to the third quartiles of the data, the horizontal lines representing the within-event regularity level median values. *p_tukey_<0.05, **p_tukey_<0.01, ***p_tukey_<0.001. b) Relationships between relative semantic similarity and memory vividness at each event regularity level. The lines and shaded bands represent linear regression lines and 95% confidence intervals. *p<0.05, **p<0.01, ***p<0.001.

We next asked if and how semantic *similarity* varied across reported events. ‘Routine’ events were overall more semantically similar to the rest of a participant’s reported events compared to ‘new’ (*b* = 0.03, *SE* = 0.006, *CI* = [0.02, 0.05], *p*_tukey_ < 0.001) and ‘periodic’ (*b* = 0.02, *SE* = 0.005, *CI* = [0.01, 0.03], *p*_tukey_ = 0.003) events (Fig. 4a). ‘Periodic’ events were more semantically similar to the rest of a participant’s reported events compared to ‘new’ events (*b* = 0.02, *SE* = 0.004, *CI* = [0.01, 0.03], *p*_*tukey*_ < 0.001). This measure validates the participant’s report of those events being ‘routine’ or ‘periodic’ because this RSS measure computes the semantic overlap across the dense sampling of experiences for each individual over a two-week period. However, although ‘new’ events were associated with the least amount of semantic overlap across experiences (had the lowest RSS overall), there were still ‘new’ events that had higher RSS compared to ‘routine’ events. Further, although ‘routine’ events had the highest RSS overall, there were ‘routine’ events that had lower RSS compared to ‘new’ events. Thus, in our next analyses we wanted to leverage this variability to examine the interaction between RSS and memory vividness across event regularity.

There was a significant main effect of event regularity level on memory vividness in our model (‘new’-’periodic’: *b* = 0.790, *SE* = 0.078, CI = [0.608, 0.972], *p*_*tukey*_ < 0.001; ‘new’-’routine’: *b* = 1.55, *SE* = 0.11, CI = [1.30, 1.80], *p*_tukey_ < 0.001; ‘periodic’-’routine’: *b* = 0.763, *SE* = 0.09, CI = [0.55, 0.97], *p*_tukey_ < 0.001), but no main effect of RSS on memory vividness when averaging across event regularity level (*b* = −0.045, *SE* = 0.53, *CI* = [−1.08, 0.994], *p* = 0.932). Critically, significant effects of RSS on memory vividness were present at independent levels of event regularity, and the directionality of that effect varied (Fig. 4b). With increasing RSS, ‘routine’ events were reported as being recalled with significantly *lower* vividness (*b* = −2.67, *SE* = 1.17, *CI* = [−4.96, −0.39], *p* = 0.022). By contrast, with increasing RSS, ‘new’ events were reported as being recalled with significantly *higher* vividness (*b* = 2.18, *SE* = 0.94, *CI* = [0.33, 4.04], *p* = 0.021). The interaction between the effect of RSS on memory vividness for ‘routine’ compared to ‘new’ events was significant (*b* = −4.86, *SE* = 1.50, *CI* = [−7.80, −1.92], *p* = 0.001). There was no significant main effect of RSS on memory vividness for ‘periodic’ events (*b* = 0.35, *SE* = 0.53, *CI* = [−0.69, 1.40], *p* = 0.506). However, the effect of RSS on memory vividness for ‘periodic’ events was significantly different from the effect of RSS on memory vividness for ‘routine’ events (*b* = 3.03, *SE* = 1.29, *CI* = [0.51, 5.55], *p* = 0.019) and was marginally different from the effect of RSS on memory vividness for ‘new’ events (*b* = −1.83, *SE* = 1.09, *CI* = [−3.96, 0.30], *p* = 0.092). This suggests that, although event regularity and RSS are significantly related measures (i.e., ‘new’ events tended to be the most semantically distinct, followed by ‘periodic’ and then ‘routine’ events), they do not produce additive effects on memory vividness. Specifically, new events that were more semantically distinct did not lead to the greatest vividness, but quite the opposite, new events with higher RSS being associated with the greatest recall vividness.

Diverging from the significant effects of RSS on memory *vividness*, we did not see that RSS had significant effects on recalled episodic *details*, both when collapsing across event regularity level (*b* = 1.00, *SE* = 1.41, *CI* = [−1.78, 3.77], *p* = 0.480), and within event regularity level (‘new’: *b* = −1.42, *SE* = 3.01, *CI* = [−7.33, 4.48], *p* = 0.636; ‘periodic’: *b* = 1.55, *SE* = 1.78, *CI* = [−1.94, 5.05], *p* = 0.384; ‘routine’: *b* = 2.25, *SE* = 3.64, *CI* = [−4.90, 9.41], *p* = 0.537). However, it should be noted again that all analyses involving recalled episodic details (and memory stability) are based on the subset of total events that were included in the event recall memory test (*N* = 565), while analyses including memory vividness include all events (*N* = 1,673), given that vividness ratings were asked for all events during the memory test. Therefore, any discrepancies between effects that involve vividness ratings compared to recalled episodic details are interpreted cautiously.

We next examined how semantic similarity influenced the stability of memory recall over time. We found that RSS did not have a significant effect on memory stability (*b* = −0.13, *SE* = 0.10, *CI* = [−0.32, 0.05], *p* = 0.165) when collapsing across event regularity level. When analyzing this relationship at each event regularity level, there were no significant effects of RSS on memory stability for ‘new’ (*b* = −0.06, *SE* = 0.20, *CI* = [−0.46, 0.33], *p* = 0.757) and ‘periodic’ (*b* = −0.07, *SE* = 0.12, *CI* = [−0.30, 0.17], *p* = 0.570) events. There was, however, a significant, *negative* relationship between RSS and memory stability for ‘routine’ (*b* = −0.51, *SE* = 0.24, *CI* = [−0.99, −0.03], *p* = 0.037) events. ‘Routine’ events that were highly semantically similar to the rest of the participant’s reported events showed significantly less memory stability (i.e., the description during the diary and recall phases diverged in their semantic content).

**Figure 5:**
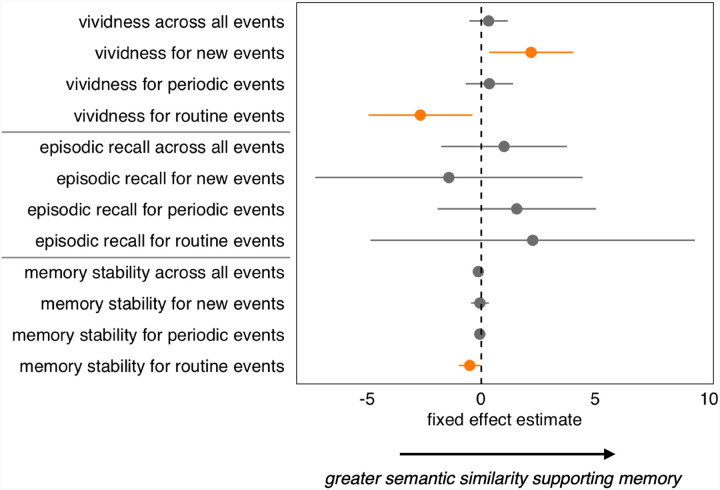
Fixed effects estimates and 95% confidence intervals are shown to summarize the effect of relative semantic similarity on different memory outcomes, the orange color indicating significant effects (*p* < 0.05) and the gray color indicating non-significant effects. The y-axis identifies the memory outcome (memory vividness, number of episodic details at recall, and memory stability) as well as event regularity level (all events, ‘new’, ‘periodic’, or ‘routine’). Horizontal lines on the y-axis distinguish outcome variables.

Taken together, event regularity and RSS demonstrated differing relationships with autobiographical memory outcomes (see Fig. 5). While event regularity was significantly associated with memory vividness (‘new’ events being the most vivid), RSS modulated this effect, decreasing vividness for ‘routine’ events, but increasing vividness for ‘new’ events. Although event regularity was significantly related to episodic detail (‘new’ events having the greatest number of episodic details at recall), RSS showed no significant relationships to recalled episodic detail. Finally, ‘new’ and ‘periodic’ events showed significantly greater memory stability compared to ‘routine’ events, while RSS did not significantly impact the memory stability of ‘new’ and ‘periodic’ events, only demonstrating a significant (negative) impact on memory stability for ‘routine’ events. These comparisons provide further evidence that these two sources of novelty in our everyday experiences, namely, participant-labeled novelty (inversely, event regularity) and relative semantic distinctiveness (inversely, RSS), have differing impacts on autobiographical memory outcomes (see Fig. 5).

### Participant-labeled novelty and relative semantic distinctiveness have differing relationships with the emotions elicited by an event

**Figure 6:**
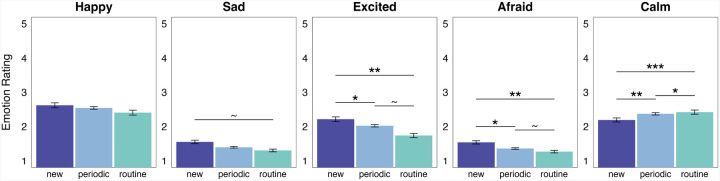
Effect of event regularity on event-level emotion ratings. Average, event-level emotion ratings and standard error bars at each event regularity level. *p_tukey_<0.05, **p_tukey_<0.01, ***p_tukey_<0.001.

Our next analyses were focused on examining how event regularity modulates emotion. For each reported event, participants rated several emotions using a Likert scale [31]. All emotion ratings were analyzed: happy, positive, sad, negative, excited, calm, and afraid. However, given that happy and positive ratings (*r*(1,671) = 0.85, *p* < 0.001), and sad and negative ratings (*r*(1,671) = 0.66, *p* < 0.001) were significantly correlated and demonstrated similar effects, only happy and sad ratings are reported here (along with excited, calm, and afraid ratings) to avoid redundancy. Event regularity had significant effects on excited, calm, and afraid ratings, while no significant relationships were found for happy and sad ratings (Fig. 6). Specifically, ‘new’ events were rated significantly higher in excitement than ‘periodic’ (*b* = 0.21, *SE* = 0.08, *CI* = [0.06, 0.37], *p*_tukey_ = 0.026) and ‘routine’ (*b* = 0.41, *SE* = 0.10, *CI* = [0.21, 0.62], *p*_tukey_ = 0.001) events. ‘New’ events were also reported with significantly higher afraid ratings compared to ‘periodic’ (*b* = 0.11, *SE* = 0.05, *CI* = [0.02, 0.20], *p*_*tukey*_ = 0.046) and ‘routine’ (*b* = 0.22, *SE* = 0.06, *CI* = [0.10, 0.34], *p*_*tukey*_ = 0.001) events. There were marginal differences in excited and afraid ratings when comparing ‘periodic’ to ‘routine’ events (excited: *b* = 0.20, *SE* = 0.08, *CI* = [0.04, 0.36], *p*_*tukey*_ = 0.618; afraid: *b* = 0.11, *SE* = 0.05, *CI* = [0.01, 0.21], *p*_*tukey*_ = 0.084). Inversely, ‘routine’ events were given significantly higher calm ratings compared to ‘periodic’ (*b* = 0.39, *SE* = 0.09, *CI* = [0.22, 0.56], *p*_*tukey*_ = 0.027) and ‘new’ (*b* = 0.19, *SE* = 0.07, *CI* = [0.04, 0.33], *p*_*tukey*_ < 0.001) events. ‘Periodic’ events were given significantly higher calm ratings compared to ‘new’ events (*b* = 0.21, *SE* = 0.06, *CI* = [0.08, 0.33], *p*_*tukey*_ = 0.003).

There was a marginal difference in sad ratings for ‘new’ compared to ‘routine’ (*b* = 0.13, *SE* = 0.06, *CI* = [0.01, 0.26], *p*_*tukey*_ = 0.090) events, and no significant differences when comparing ‘periodic’ to ‘new’ (*b* = −0.08, *SE* = 0.05, *CI* = [−0.17, 0.01], *p*_tukey_ = 0.169) and ‘routine’ (*b* = 0.05, *SE* = 0.05, *CI* = [−0.05, 0.15], *p*_tukey_ = 0.614) events. The effect of event regularity on happy ratings (which was tested using a multi-level model with full random effects) produced no significant or marginal differences (‘new’ vs ‘periodic’: *b* = −0.10, *SE* = 0.09, CI = [−0.27, 0.07], *p*_*tukey*_ = 0.509; ‘new’ vs ‘routine’: *b* = −0.16, *SE* = 0.12, *CI* = [−0.39, 0.08], *p*_*tukey*_ = 0.414; ‘periodic’ vs ‘routine’: *b* = −0.06, *SE* = 0.09, *CI* = [−0.23, 0.12], *p*_tukey_ = 0.811). Thus, we found that individual emotion ratings, for the most part, did vary as a function of event regularity. However, only excited, afraid, and calm ratings showed significant differences depending on event regularity level.

**Figure 7:**
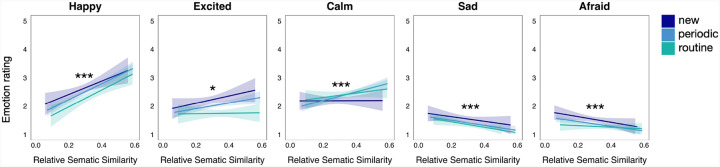
Effect of relative semantic similarity on event-level emotion ratings. The lines and shaded bands represent linear regression lines and 95% confidence intervals. *p<0.05, **p<0.01, ***p<0.001. Significance level is based on main effects (i.e., collapsing across event regularity level). Within-event regularity regression lines are shown for visual interpretation.

We next asked how RSS was related to reported emotions experienced during each event (Fig. 7). All multi-level models were able to run without overfitting warnings when using a full random effects structure, except for the model predicting afraid ratings, which was run using a random-intercepts-only structure. We found that RSS predicted significantly higher happy (*b* = 3.45, *SE* = 0.42, *CI* = [2.61, 4.28], *p* < 0.001), excited (*b* = 1.04, *CI* = [0.29, 1.80], *p* = 0.007), and calm (*b* = 2.76, *CI* = [2.02, 3.49], *p* < 0.001) ratings when collapsing across event regularity level. Further, greater RSS predicted significantly lower sad (*b* = −1.35, SE = 0.28, *CI* = [−1.90, −0.80], *p* < 0.001) and afraid (*b* = −1.31, *CI* = [−1.81, −0.82], *p* < 0.001) ratings when collapsing across event regularity level. When analyzing these relationships within event regularity level, effects remained significant at each event regularity level except for the effect of RSS on sad ratings for ‘new’ events and the effect of RSS on excited and afraid ratings for ‘routine’ events (see Supplementary Materials for more details).

In sum, similar to the impacts found on memory, event regularity and RSS had differing relationships with the emotions experienced during the event. New events were associated with the greatest arousal (i.e., greater excitement and fear, less calmness), but did not elicit significantly greater happiness or sadness compared to periodic and routine events. This suggests that event regularity may significantly modulate arousal, but not valence. Alternatively, increasing RSS generally supported positive emotions (i.e., happiness, excitement, and calmness), while minimizing negative emotions (i.e., sadness and fear).

### Relative emotional distinctiveness predicts greater episodic memory: vividness and episodic detail

Critically, not only can the content of autobiographical experiences be relatively novel, but the feelings elicited by those experiences. Therefore, multivariate emotion signatures were calculated using the participants’ own event-level emotion ratings (RES; see Methods) to test whether the relative peaks and valleys in their ‘emotional landscapes’ were remembered more vividly.

First, we tested the relationship between relative emotion similarity (RES) and event regularity, and additionally, RES and RSS. ‘New’ events were associated with more distinct emotional patterns (had significantly lower RES) compared to ‘periodic’ (*b* = −0.02, *SE* = 0.005, *CI* = [−0.02, −0.01], *p*_*tukey*_ < 0.001) and ‘routine’ (*b* = −0.03, *SE* = 0.006, *CI* = [−0.04, −0.02], *p*_*tukey*_ < 0.001) events (Fig. 7a). ‘Periodic’ events had marginally distinct emotional patterns compared to ‘routine’ events (*b* = −0.01, *SE* = 0.005, *CI* = [−0.02, 0.00], *p*_*tukey*_ = 0.068). Using a full random effects structure, greater relative emotional distinctiveness significantly predicted greater relative semantic distinctiveness (*b* = 0.15, *SE* = 0.04, *CI* = [0.08, 0.22], *p* < 0.001) when collapsing across event regularity level. At each event regularity level, there was a marginal effect of RES on RSS for ‘new’ events (*b* = 0.11, *SE* = 0.06, *CI* = [0.00, 0.23], *p* = 0.057), a significant effect of RES on ‘periodic’ events (*b* = 0.16, *SE* = 0.04, *CI* = [0.08, 0.24], *p* < 0.001), and no significant effect of RES on ‘routine’ events (*b* = 0.07, *SE* = 0.07, *CI* = [−0.08, 0.21], *p* = 0.363). Notably, the effect sizes are small, which suggests that RES and RSS are related, but are not capturing the exact same information.

Knowing that RES was related to event regularity and RSS, the impact of RES on the memory measures was analyzed next. There was a main effect of RES on memory vividness (Fig. 7b): events that were experienced with greater emotional distinctiveness were given higher vividness ratings at recall (*b* = −1.64, *SE* = 0.47, *CI* = [−2.57, −0.71], *p* < 0.001). When analyzing this relationship at each event regularity level, there was no significant effect of RES on memory vividness for ‘new’ (*b* = −1.42, *SE* = 0.97, *CI* = [−3.32, 0.48], *p* = 0.143) or ‘routine’ events (*b* = −1.79, *SE* = 1.19, *CI* = [−4.12, 0.55], *p* = 0.133), and a significant, negative effect of RES on ‘periodic’ events (*b* = −1.69, *SE* = 0.61, *CI* = [−2.89, −0.49], *p* = 0.006), meaning that more emotionally distinct ‘periodic’ events were recalled with greater vividness.

Greater emotional distinctiveness also significantly predicted greater recalled episodic details when collapsing across event regularity level (*b* = −4.47, *SE* = 1.53, *CI* = [−7.48, −1.47], *p* = 0.004). Within event regularity level, this relationship remained significant for ‘new’ (*b* = −7.84, *SE* = 3.22, *CI* = [−14.16, −1.52], *p* = 0.015) and ‘periodic’ (*b* = −4.86, *SE* = 2.00, *CI* = [−8.79, −0.94], *p* = 0.015) events, but not for ‘routine’ (*b* = 0.69, *SE* = 3.47, *CI* = [−6.13, 7.50], *p* = 0.843) events. RES did not significantly predict memory stability when collapsing across event regularity level (*b* = −0.14, *SE* = 0.10, *CI* = [−0.35, 0.06], *p* = 0.166), or within each event regularity level for ‘new’ (*b* = −0.04, *SE* = 0.22, *CI* = [−0.46, 0.39], *p* = 0.855) and ‘periodic’ (*b* = −0.10, *SE* = 0.13, *CI* = [−0.36, 0.17], *p* = 0.464) events. There was, however, a marginal, positive effect of emotional distinctiveness on memory stability for ‘routine’ events (*b* = −0.40, *SE* = 0.23, *CI* = [−0.86, 0.06], *p* = 0.087). These results suggest that emotional distinctiveness positively impacts memory vividness and episodic details at recall, and has a marginal, positive effect on the memory stability for ‘routine’ events.

## Discussion

In the current study, we aimed to explore how variation in event novelty impacted long-term measures of memory recall. We found that participant-labeled novelty (i.e., a ‘new’ event) and emotional distinctiveness increase memory vividness and episodic detail at recall. Participant-labeled novelty was uniquely associated with greater memory stability across time. A second major finding, however, was that memory vividness for ‘new’ events benefitted from greater relative semantic *similarity* (i.e., a familiarity ‘scaffold’), rather than more ‘maximal’ novelty.

The ‘Predictive Interactive Multiple Memory Signals’ (PIMMS) [32, 33] model outlines why the presence of both novelty and semantic similarity may benefit memory more than maximal novelty. According to this PIMMS model, ‘maximal’ novelty, a scenario in which an individual encounters unknown objects in an unknown environment, may produce minimal learning given the lack of prior experience to reference. Critically, our results provide insight as to how this predictive model maps onto participants’ day-to-day real-world experiences and include important variance, given that we utilize continuous measures and different types of novelty. This approach provided us the opportunity to demonstrate what specific compositions of novelty and congruency may produce the greatest possible episodic recall. In line with the PIMMS model, our results show that ‘new’ events with a stronger semantic scaffold (higher RSS), which may serve as an informative prior, are later remembered with greater vividness than ‘new’ events that lack a semantic scaffold (lower RSS), suggesting a weak or flat prior (and therefore a small or nonexistent prediction error, which leads to minimal learning).

However, it should be emphasized that, in our study, new events that were low in relative semantic similarity (more ‘maximally’ novel) were still remembered more vividly than ‘periodic’ and ‘routine’ events, showing that there is still some memory benefit related to more maximal, or additive, novelty. Prior work has suggested that memory for absolute novelty and contextual novelty may rely on distinct hippocampal-related processes [34, 35]. There is also evidence showing a gradual decline in hippocampal activity during continued novelty exposure [36–38], suggesting that the relationship between novelty and hippocampal response changes as a function of context stability, or familiarity. Bein and colleagues [39] have outlined various neural mechanisms that may be recruited depending on how incoming information relates to prior knowledge. Future neuroimaging work should be conducted to investigate how the relative semantic similarity of novel autobiographical events impacts which neural mechanism is used, and how that in turn is related to different episodic memory outcomes.

It will also be important to investigate whether different types of novelty (participant-labeled novelty, semantic novelty, and emotional novelty) modulate memory at distinct stages in the life of a memory. It is known that incoming novel information benefits from hippocampal engagement during encoding, supporting the formation of an episodic memory trace [40–42]. Research has shown that when this novel information aligns with pre-existing schemas, it is more readily integrated into neocortical memory networks, such as the medial prefrontal cortex (mPFC), which facilitates rapid consolidation [12, 13] and stabilizes memory traces [43]. Work has also demonstrated that the magnitude of post-encoding connectivity between the hippocampus and mPFC is related to the long-term structured representation of schematic knowledge in the mPFC [44, 45]. Finally, based on prior work that utilized a variety of approaches (i.e., imaging, mouse genetics, pharmacological and anatomical lesions, testing both humans and animals with mPFC damage) a model has been proposed suggesting that, during successful delayed recall, the hippocampus and the mPFC interact. More specifically, the mPFC supports the retrieval of goal-relevant information and the hippocampus reconstructs episodic content [46–48]. Our findings suggest that ‘new’ events may enhance hippocampal encoding allowing for the formation of distinct, episodic memory traces, while semantic similarity (RSS) may reflect mPFC engagement and schema-based consolidation. Another non-mutually exclusive possibility is that post-encoding hippocampal reactivation will prioritize novel events while cortical reactivation will be enhanced for novel experiences that contain more of a semantic scaffold (high RSS). Recent empirical work has shown that cortical, but not hippocampal, reactivation is increased for events that are repeated [49]. Focusing on emotional distinctiveness (RES), this type of novelty may primarily engage the arousal system at encoding, triggering neuromodulatory responses that prioritize memory [50] and initiate ‘tag- and-capture’ processes [9]. Emotional distinctiveness may recruit the amygdala, which has been shown to support durable emotion–item associations, thus making them more resistant to forgetting [51]. Although difficult to gather neural data during naturalistic experience, future work can aim to scan participants during the recall of these distinct events to test these hypotheses above which are based on a wealth of controlled laboratory studies.

Although memory vividness, episodic detail at recall, and memory stability were significantly related memory outcomes, some of our results demonstrated apparent divergences between the effects of novelty on these memory outcomes. However, this should be interpreted cautiously at this time, given that we had reduced power for episodic details and memory stability (due to about one-third of the events having corresponding recalls). Future work will need to investigate whether the effects regarding episodic detail and memory stability found in this study persist with a greater sample of event recalls, or the effects change due to our sample size being underpowered.

Shifting from memory outcomes to our emotion outcomes, participant-labeled novelty differentially modulated emotions associated with arousal, while relative semantic *similarity* supported positive emotions and minimized negative emotions. This suggests that a relative scaffolding of familiarity may not only benefit memory, but relates to emotional well-being. This finding is reminiscent of the ‘mere-exposure effect’, which demonstrates that repeated exposure to a stimulus (i.e., familiarity) can increase positive affect or ‘liking’ of that stimulus [52, 53]. This ‘liking’ of familiarity relates to prior animal behavior work [54], rats having demonstrated a preference for a familiar over a novel stimulus when first exposed to a novel environment, then preferred the novel stimulus once they were habituated to the environment. These rats additionally changed their preference back to the familiar stimulus after just being exposed to a different novel environment. This suggests that rats prefer a degree of relative familiarity, rather than ‘maximal’ novelty.

Interestingly, our findings diverge from prior work on real-world novelty and emotion. Previous studies found that greater spatial exploration [55, 56] and greater event-level composite uniqueness [19] were associated with greater positive affect. An important distinction is that those studies investigated mood once at the end of each day (day-level), while we analyzed emotions for each reported event (event-level). To attempt to tease apart this distinction and given that our participants also provided day-level mood ratings, we tested how daily positivity ratings related to novelty. Indeed, we found a significant, positive effect of novelty on day-level positivity (*b* = 0.22, *SE* = 0.08, *CI* = [0.07, 0.37], *p* = 0.005): participants reported higher positivity at the end of the day when they also reported at least one new event. These findings suggest that although event-level novelty may not be linked to more positive emotions directly, people may generally feel happier on days when they did something new. Future work is needed to better understand these effects.

## Conclusion

Collectively, the findings from this study provide a more nuanced understanding as to what composition of novelty may produce the greatest episodic recall. Compared to ‘maximal’ novelty, the interplay of novelty and congruency may produce greater episodic memory for autobiographical events, which we term the ‘something old, something new’ principle. Further, our finding that a semantic scaffold further boosts memory vividness for new events supports the consideration of ‘absolute novelty’ and ‘absolute congruency’ as two ends of a continuous spectrum, rather than separate, binary factors, especially when analyzing autobiographical data. Our work also highlights how the operationalization of novelty can impact its relationship to memory and emotion, which may assist in disentangling potential conflicting findings in novelty and memory research. Additionally, collecting and subsequently testing memory for autobiographical events using a daily diary paradigm (i.e., utilizing a prospective memory design) provides an exemplar of each participant’s day-to-day experiences, and in our case, the emotions associated with those experiences. Such data are well-suited for NLP techniques, which have only become a recent possibility with much to be explored [57]. Our results, as well as other recent work [58–60], provide confidence that combining dense-sampling paradigms with NLP-driven analyses are valuable trails worth trekking, all in pursuit of better understanding the complex, multidimensional stories that provide measurement and depth to our lives.

## Supplementary Material

Supplementary Files

This is a list of supplementary files associated with this preprint. Click to download.

- SupplementaryMaterials.pdf

## Figures and Tables

**Figure 8:**
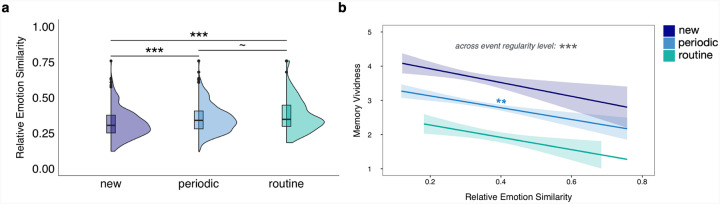
Effect of event regularity on relative emotion similarity, and relative emotion similarity on memory vividness. a) The half-violin plots represent the overall distributions of relative emotion similarity values at each event regularity level. The boxplots within the half-violin plots span from the first to the third quartiles of the data, the horizontal lines representing the within-event regularity median values. *p_tukey_<0.05, **p_tukey_<0.01, ***p_tukey_<0.001. b) Relationships between relative emotion similarity and memory vividness at each event regularity level. The lines and shaded bands represent the linear regression lines and 95% confidence intervals. *p<0.05, **p<0.01, ***p<0.001.

**Figure 9:**
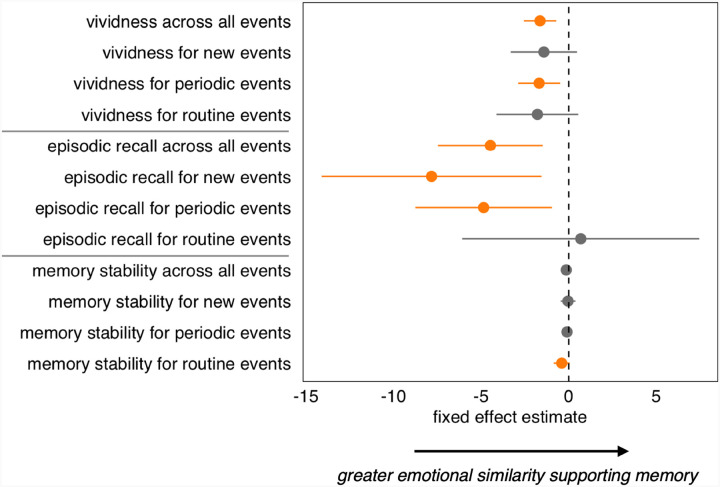
Fixed effects estimates and 95% confidence intervals are shown to summarize the effect of relative emotion similarity on different memory outcomes, the orange color indicating significant effects (*p* < 0.05) and the gray color indicating non-significant effects. The y-axis identifies the memory outcome (memory vividness, number of episodic details at recall, and memory stability) as well as event regularity level (all events, ‘new’, ‘periodic’, or ‘routine’). Horizontal lines on the y-axis distinguish outcome variables.

## Data Availability

The dataset analyzed during the current study is available from the corresponding author (eew2153@columbia.edu) on reasonable request.
